# Genotypes of *Sechium* spp. as a Source of Natural Products with Biological Activity

**DOI:** 10.3390/life15010015

**Published:** 2024-12-27

**Authors:** Jorge Cadena-Iñiguez, Ma. de Lourdes Arévalo-Galarza, Edelmiro Santiago-Osorio, Itzen Aguiñiga-Sánchez, Jorge David Cadena-Zamudio, Marcos Soto-Hernández, Yeimy C. Ramírez-Rodas, Lucero del Mar Ruiz-Posadas, Sandra Salazar-Aguilar, Víctor Manuel Cisneros-Solano

**Affiliations:** 1 Colegio de Postgraduados, Campus San Luis Potosí, San Luis Potosí 78620, San Luis Potosí, Mexico; 2 Interdisciplinary Research Group at Sechium edule in Mexico, A.C., Agustin Melgar 10, Col. Niños Héroes, Texcoco 56160, Estado de México, Mexico; 3 Programa de Botánica y Fisiología, Colegio de Postgraduados, Campus Montecillo, Texcoco 56264, Estado de México, Mexico; 4 Hematopoiesis and Leukemia Laboratory, Research Unit on Cell Differentiation and Cancer, Universidad Nacional and Autónoma de México, FES Zaragoza, Mexico City 09230, Mexico; 5 Department of Biomedical Sciences, School of Medicine, Universidad Nacional Autónoma de México, FES Zaragoza, Mexico City 09230, Mexico; 6 Instituto Nacional de Investigaciones Forestales, Agrícolas y Pecuarias, Centro Nacional de Recursos Genéticos, Blvd. de la Biodiversidad #400 Rancho las Cruces, Tepatitlán de Morelos 47600, Jalisco, Mexico; 7 Instituto Nacional de Investigaciones Forestales, Agrícolas y Pecuarias, Valles Centrales, C. Melchor Ocampo 7, Sto. Domingo Barrio Bajo, Etla, Oaxaca 68200, Oaxaca, Mexico; 8 Centro Regional Universitario Oriente, Universidad Autónoma Chapingo, Huatusco 94100, Veracruz, Mexico

**Keywords:** phytogenetic resources, conservation of genotypes, secondary metabolites, biological activity, extraction, yields

## Abstract

The genus *Sechium* P. Br. (Cucurbitaceae) includes ten species, two of which are edible. The inedible genotypes are in a fragile ecological niche, since they are not used by rural inhabitants. A rescue and genetic crossing program was designed to identify uses that favor their conservation due to their content of bioactive secondary metabolites (Sm) for health. Fruits of *S. compositum* (wild type), hybrid H-D Victor (inedible), and *S. edule* var*. nigrum spinosum* (edible) were evaluated by extraction methods such as juice and oven drying to determine the yields of Sm, with in vivo evaluations of liver damage. The dried biomass (40 °C) extracted with ethanolic and methanolic procedures showed lower Sm content than the juice (fresh biomass). More than 90% of phenolic acids and cucurbitacins in the extracts were degraded, possibly due to the drying time (oven). Biological activity showed that *nigrum spinosum* and HD-Victor have fewer toxic metabolites than *S. compositum*. The hybrid H-D Victor is of reduced cytotoxicity, showing the advantages of hybridization with wild types. Phytochemical and biological activity characterization may contribute to the conservation of genotypes and become a source of bioactive natural products.

## 1. Introduction

Agrobiodiversity for food and agriculture is a crucial component of biodiversity, encompassing cultivated plants and domesticated animals in agricultural, forestry, and aquaculture systems [1]. Mexico stands out globally as the fifth most biodiverse country in plants and the sixth in endemism [2]. The diversity of vascular plants in Mexico ranges from 22,000 to 31,000 species, with around 4000 used medicinally and 1000 to 1500 considered edible [3,4]. As a domestication hub within Mesoamerica, Mexico is recognized as a Vavilov center, crucial for global food systems. It is the center of origin and diversity for key crops such as maize, beans, cacao, and vanilla, among others [5,6].

However, Plant Genetic Resources for Food and Agriculture (PGRFA) have sharply declined, particularly in tropical regions, due to factors such as agricultural intensification, deforestation, land use changes, privatization of genetic resources, and insufficient research on new applications [7]. This erosion reduces options to ensure food sovereignty and adapt agriculture to climate change. The Second Global Plan of Action for PGRFA [8] addresses this issue by prioritizing conservation, sustainable use, and capacity building through both in situ and ex situ methods. It also emphasizes the characterization and evaluation of genetic resources to prevent underutilization, encouraging phytochemical characterization and functional activity assessment to support conservation and utilization [1]. In this context, the characterization of PGRFA aims to fill information gaps and enhance access to data in gene banks, expanding the pool of resources available for new or optimized uses [1,9]. This effort aligns with the bioeconomy, which promotes the production, use, and conservation of biological resources, supported by science, technology, and innovation, to develop sustainable products, processes, and services [10]. This approach enhances agricultural sustainability by optimizing biological and genetic resources while supporting biotechnologies that diversify applications [11]. Morphometric and phytochemical characterizations are essential initial steps to evaluate the functional potential of these resources.

In this context, the genus *Sechium* P. Br. exemplifies the diversity and utility of PGRFA, encompassing eight wild non edible species (*S. chinantlense*, *S. compositum*, *S. hintonii*, *S. talamancense*, *S. panamense*, *S. pittieri*, *S. venosum*, and *S. vilosum*) and two cultivated species (*S. tacaco* and *S. edule*), with *S. edule* being the most nutritionally significant due to the consumption of all its parts. This species shows extensive morphological variability, reflected in detailed classifications of its genotypes. Yellow-skinned fruit with or without spines genotypes include *albus minor*, *a. levis*, *a. dulcis*, and *a. spinosum*, while green-skinned genotypes include *nigrum minor*, *n. conus*, *n. maxima*, *n. levis*, *n. spinosum*, *n. xalapensis*, and *virens levis*. Additionally, there is a bitter genotype, *amarus sylvestris* [12,13,14]. All *Sechium* genotypes produce secondary metabolites (Sm), but bitter genotypes have significantly higher levels of tetracyclic triterpenes, with edible varieties containing ten to one hundred times fewer of these compounds. This disparity is linked to habitat adaptations and the impacts of cultivation and ancestral unplanned selection [8,9]. The Germplasm Bank collection has facilitated the development of new *Sechium* genotypes as food sources and for their biologically active metabolites, which are now undergoing phytochemical and pharmacokinetic evaluations in a genetic improvement program (Table 1) [15]. Among chayote’s most notable properties are its antiproliferative effects, demonstrated across genotypes such as *virens levis*, *nigrum spinosum*, Perla negra, and the hybrid H-387-07-GISeM. These genotypes exhibit potent cytotoxicity against cancer cell lines including HeLa, L929, P388, MCF7, J774, and WEHI-3, with IC_50_ values below 1.3 µg/mL in some cases. Mechanisms include inhibition of DNA synthesis, induction of apoptosis, and disruption of oxidative pathways. The methanol extract of Perla negra and the dichloromethane extract of Madre Negra showed strong activity against HeLa and MCF7 cells, respectively, with results comparable to doxorubicin. Similarly, the H-387-07-GISeM hybrid extract induced apoptotic markers such as phosphatidylserine translocation and DNA fragmentation, selectively targeting leukemia cells while sparing normal bone marrow cells. These effects are primarily attributed to cucurbitacins and flavonoids present in active fractions, highlighting the therapeutic potential of chayote genotypes as safe and effective antitumor agents [16,17,18,19,20]. Beyond its anticancer properties, chayote provides significant benefits for managing metabolic syndrome (MetS). A six-month supplementation study with the *nigrum spinosum* variety in older adults revealed reductions in oxidative stress markers, such as lipoperoxides and 8-isoprostanes, along with improvements in HbA1c levels, HDL cholesterol, and anti-inflammatory cytokine IL-10. These findings highlight its antioxidant, anti-inflammatory, hypotensive, and hypoglycemic effects, positioning chayote as a promising nutraceutical intervention for MetS [21]. In addition, to further enhance its therapeutic potential, innovative delivery methods like microencapsulation have been developed. Encapsulated extracts of Perla negra retained their antiproliferative efficacy, offering prolonged release and stability. This advancement underscores chayote’s applicability in both clinical and industrial settings, solidifying its role as a versatile and effective natural therapeutic agent [19]. In conclusion, the diverse genotypes of chayote exhibit a wide range of bioactivities closely linked to their secondary metabolite composition, representing a versatile and valuable resource for developing nutraceuticals and pharmacological agents. These findings pave the way for further exploration of chayote’s therapeutic potential and its applications in functional foods and medicine. Due to the above, the selection of *Sechium compositum*, *Sechium edule* var. *nigrum spinosum*, and the hybrid HD-Victor was based on their distinct biochemical and ecological characteristics. *Sechium compositum* represents a non-edible wild species rich in secondary metabolites, *S. edule* var. *nigrum spinosum* is a cultivated variety with unique metabolomic traits, and HD-Victor offers a hybrid perspective combining traits of wild and cultivated germplasm. Due to these characteristics, the aim of this study is to analyze the phytochemical content of the fruits of two bitter *Sechium* genotypes and one edible species (Cucurbitaceae), with a focus on the differences between extraction methods, metabolite yield, and their potential biological activity.

## 2. Materials and Methods

### 2.1. Sechium Compositum

**Figure 1:**
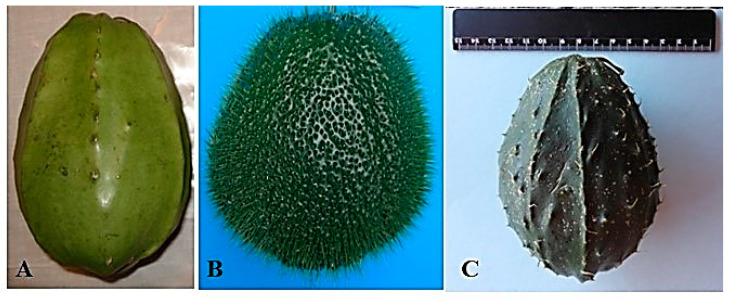
Fruits of (**A**): *Sechium compositum*, (**B**): *Sechium edule* var*. nigrum spinosum*, and (**C**): Hybrid HD-Victor (The scale used corresponds to a metric ruler, where each number represented is equivalent to 1 cm).

Horticulturally mature fruits (20 ± 2 days after anthesis) of the non-edible *Sechium compositum* were selected from the *Sechium* P. Br. germplasm bank collection. These genotypes are part of a breeding program aimed at developing new varieties and hybrids (Table 1), which serve as sources for nutraceutical foods, food ingredients, and biologically active compounds. These compounds have potential applications in the treatment of public health concerns, including cancer (antineoplastics), antioxidants, antidiabetics, and therapies for metabolic syndrome (Figure 1A). The Germplasm Bank is located in Mexico (19°08′48″ N and 97°57′00″ W), within the Mesophilic Mountain Forest, a region characteristic of cloud forest vegetation at an altitude of 1340 m above sea level (masl). The area has an average annual temperature of 19 °C and a relative humidity of 85%, and receives an annual precipitation of 2250 mm. The soils in this region are nutrient-rich vitric luvisols with a coarse texture, containing volcanic glass fragments. These soils are moderately fertile, ranging from slightly acidic to acidic pH (4.3–6.5), and are rich in organic matter, with low calcium levels but high concentrations of iron, manganese, and zinc [15]. All the genotypes evaluated were developed and grown under identical agroclimatic conditions, ensuring uniformity in their development and subsequent evaluation.

### 2.2. Obtaining Methanolic and Ethanolic Extracts from Sechium Compositum Fruits

Methanolic and ethanolic extraction methods were applied to the juice of *Sechium compositum*. The fruits were first cut and dried at 45 °C in a convection oven (RIOSSA E-33, Mexico, Mexico) until a constant weight was achieved. The dried material was then standardized using an electric mill to obtain small particles, which were stored in glass jars in a cool, dry place. A discontinuous extraction process was carried out as follows: 500 g of ground plant material was placed in separate 1.0 L flasks with methanol (99.8%, J.T. BAKER, Phillipsburg, NJ, USA) and ethanol (absolute ethyl alcohol, J.T. BAKER). The flasks were left to stand for 48 h at room temperature (20 °C). After this period, the solvent was replaced, and the supernatant was filtered. The solvent was then evaporated using a rotary evaporator at 45 °C. This procedure was repeated until the extracts no longer exhibited a green color, a clear indication of the removal of chlorophylls. Chlorophylls, being highly soluble in polar organic solvents such as methanol and ethanol, are among the first pigments to be extracted during plant material processing, imparting a characteristic green coloration to the solution [22,23]. The loss of green color during successive extractions reflects the progressive depletion of these pigments from the plant matrix, which is a widely accepted preliminary indicator in extraction protocols [24]. While the absence of green pigmentation was used here as a qualitative criterion to monitor chlorophyll removal, this step also ensured that subsequent analyses were not confounded by the presence of these pigments, which could interfere with the isolation and characterization of bioactive compounds. By repeating the extraction process until decolorization was achieved, we aimed to maximize the specificity of the extracts for compounds of interest, a standard approach in phytochemical studies [23,24].

### 2.3. Obtaining Juice

To obtain the juice, *Sechium compositum* fruits were cut into small pieces. The juice was then extracted using an OPM-500 industrial processor (JOOSHUN™, China) and subsequently centrifuged at 2150 g for 10 min. The resulting supernatant was stored at 4 °C for later use.

### 2.4. Identifying Phenolic Acids by High Performance Liquid Chromatography (HPLC)

An Infinity 1100 chromatograph from Agilent Technologies (St. Clara, CA, USA) was used under the following conditions: a Nucleosil^®^ 5 μm C18 100 Å column (125 × 4 mm) from Macherey-Nagel, with a gradient of solvent (A) water (pH 2.5) containing trifluoroacetic acid (TFA) and solvent (B) acetonitrile (ACN). The flow rate was set at 1.0 mL/min, with the column maintained at 30 °C. The injection volume was 20 μL, and the system operated at a pressure of 114 bar. The inclusion of TFA in solvent (A) was crucial to adjust the pH of the mobile phase, thereby influencing the ionization state of analytes and enhancing their retention and resolution [25]. Furthermore, TFA protonates silanol groups on the stationary phase, minimizing unwanted interactions that can cause peak tailing and improving overall peak symmetry [26,27,28]. Its volatility also ensures compatibility with mass spectrometry, as it leaves no non-volatile residues [27,28]. The following acids were used as standards: gallic, chlorogenic, syringic, vanillic, *p*-hydroxybenzoic, caffeic, ferulic, and *p*-coumaric. The parameters for method validation, including the calibration equations, limits of detection (LODs), limits of quantification (LOQs), retention times, and recovery percentages of the analytes, were determined based on the experimental design and HPLC conditions described in this study (Tables S1–S4).

### 2.5. Flavonoids

Flavonoid analysis was conducted at 30 °C using a Hypersil™ ODS-2 column (125 × 4 mm) with a 5 μm particle size. The mobile phase consisted of a water mixture (65:35 *v*/*v*), with the water’s pH adjusted to 2.5 using trifluoroacetic acid. The chromatographic conditions were set as follows: a flow rate of 1.0 mL/min, a sample injection volume of 20 µL, and a system pressure of 144 bar. The compounds used as standards for this analysis were rutin, phloridzin, myricetin, quercetin, naringenin, phloretin, and galangin.

### 2.6. Cucurbitacins

Cucurbitacins were analyzed at 30 °C using a Hypersil™ ODS-2 column (125 × 4 mm) with a 5.0 μm particle size. The mobile phase consisted of a water mixture (65:35 *v*/*v*), with the water’s pH adjusted to 2.5 using trifluoroacetic acid. The chromatographic conditions were as follows: a flow rate of 1.0 mL/min, a sample injection volume of 20 µL, and a system pressure of 144 bar.

### 2.7. Statistical Analysis

The secondary metabolite content of the extracts and juice was analyzed using a completely randomized design with three repetitions. An analysis of variance (ANOVA) and Tukey’s tests (*p* < 0.05) were conducted to compare the results, utilizing SAS^®^ Institute 9.0 software.

### 2.8. Obtaining Extracts from a HD-Victor Hybrid and S. edule *var.* nigrum spinosum

Fresh, horticulturally mature fruits (18 ± 2 days after anthesis) were used to evaluate the biological activity, specifically the hepatoprotective effect of *Sechium edule* var. *nigrum spinosum* (Figure 1B) and the HD-Victor hybrid (Figure 1C) [16]. The environmental and soil conditions were consistent for both genotypes, as described in previous sections [15]. The fruits were washed, dried, weighed, and cut into flakes, including the exocarp, mesocarp, spines, and seed. The flakes were then dried at 45 °C in an oven with air circulation (BLUE-M Electronic Company, Blue Island, IL, USA) until fully dehydrated. Afterward, the dried flakes were ground to a particle size of 2 mm.

### 2.9. Extraction Process for HD-Victor Hybrid and S. edule *var.* nigrum spinosum

The initial weight of the fresh fruits was 80 kg for var. *nigrum spinosum* and 15 kg for the HD-Victor hybrid, yielding 5.24 kg and 1.56 kg of dry material, respectively. A discontinuous chemical extraction was performed for each sample, using 1.5 kg of the ground material macerated in methanol at room temperature (22 ± 2 °C). The solvent was then replaced, the mixture was filtered using Whatman No. 1 paper, and the solvent was evaporated at 45 °C using a Büchi R-114 rotary evaporator (Büchi, Flawil, Switzerland).. The extract was then concentrated and recovered. This process was repeated 25 times until the solvent became colorless [21,29]. The resulting extracts were stored in amber bottles at room temperature.

### 2.10. Identification of Secondary Metabolites by High Performance Liquid Chromatography (HPLC)

A total of 40 mg of each extract was weighed and diluted in 2.0 mL of HPLC-grade methanol (Sigma-Aldrich, St. Louis, MO, USA)). The solution was then filtered through a 0.22 µm membrane (Millipore, Cork, Ireland) and placed in HPLC vials. Each extract and its corresponding groups of metabolites were analyzed by High-Performance Liquid Chromatography (HPLC). Cucurbitacins were analyzed using a Symmetry Shield C18 Column (4.6 × 250 mm) ((Waters, Cerdanyola del Vallès, Spain) by isocratic analysis. The mobile phase consisted of a water:methanol mixture (50:30:20), with a flow rate of 1.0 mL/min, a pressure of 179 bar, a temperature of 25 °C, and an injection volume of 20 µL. The detection wavelengths were set at λ1 235 nm and λ2 254 nm, and the analysis time was 60 min. Cucurbitacins E, I, B, and D (Sigma-Aldrich, St. Louis, MO, USA) were used as reference standards. For phenolic acids, the analysis was performed using a Nucleosil 100 Å column (125 × 4.0 mm, Hewlett-Packard, Palo Alto, CA, USA) in a binary solvent system. The solvents used were (A) water with a pH of 2.5 adjusted with trifluoroacetic acid (TFA), and (B) acetonitrile (ACN). The conditions were as follows: a flow rate of 1.0 mL/min, pressure of 106 bar, column temperature of 30 °C, injection volume of 20 µL, detection wavelength at λ1 280 nm, and a 30 min analysis time. The phenolic acid standards used were gallic, ferulic, *p*-hydroxybenzoic, caffeic, *p*-coumaric, vanillic, chlorogenic, and syringic acids. For flavonoid analysis, the samples were incubated at 70 °C for one hour and then allowed to cool before being analyzed on a Hypersil ODS column (125 × 4.0 mm, Hewlett-Packard) in a binary system. The solvents were (A) water with pH adjusted to 2.5 with TFA, and (B) acetonitrile. A DAD detector was used, and the conditions were a flow rate of 1.0 mL/min, pressure of 106 bar, an injection volume of 5.0 µL, detection wavelengths of λ1 316 nm, λ2 365 nm, and λ3 254 nm, a column temperature of 30 °C, and a 25 min analysis time. The flavonoid standards used were rutin, phloridzin, myricetin, quercetin, naringenin, phloretin, apigenin, and galangin. Limits of quantification (LOQs), retention times, and recovery percentages of the analytes were determined based on the experimental design and HPLC conditions described in this study (Tables S1–S4).

#### Extract Preparation for Biological Activity

The extracts obtained under the previously described conditions were weighed and dissolved in a phosphate buffer solution (PBS). Afterward, they were sterilized using UV light at 280 nm for 20 min and stored at 4 °C until administration.

### 2.11. Biological Activity

Female and male 2.5-month-old CD-1 strain mice (*Mus musculus* L.), obtained from the animal facility of the Faculty of Higher Studies Zaragoza-UNAM, were housed under controlled conditions. They were maintained on a 12 h light–dark cycle, with an average temperature of 22 °C, and provided with sterile water and standard food. The bioassays were conducted following the animal care and husbandry guidelines established in the Official Mexican Standard (NOM-062-ZOO-1999), which outlines the technical specifications for the production, care, and use of laboratory animals. The extracts were administered to the mice via intraperitoneal injections at varying doses.

To evaluate acute toxicity, the method previously described [30] was followed. In brief, both female and male CD-1 mice, kept under the aforementioned conditions, were divided into 12 groups. Twenty-four hours prior to the trial, the mice were fasted. Afterward, various concentrations (0, 8, 16, 40, 160, 400, 600, 1600, 2900, and 5000 mg/kg of body weight) were administered, and the mice were monitored at 1, 2, 3-, 6-, 12-, and 24-h post-treatment. Mortality was recorded over a seven-day period. The data were analyzed using probit analysis to calculate the LD_50_, with the IBM SPSS Statistics program (version 20; Corporation, Chicago, IL, USA). The P388 cell line, derived from mice with macrophagic leukemia, was obtained from the American Type Culture Collection (ATCC: The Global Bioresource Center, Manassas, VA, USA). The cell lines were cultured in Iscove’s Modified Dulbecco’s Medium (IMDM) from GIBCO-BRL Invitrogen (Grand Island, NY, USA), supplemented with 10% deactivated fetal bovine serum (Invitrogen GIBCO-BRL, Grand Island, NY, USA). The cells were maintained in 5.0 mL glass Petri dishes (Pyrex, Corning, NY, USA) at 37 °C in an incubator (Thermo Forma, Marietta, OH, USA) under an atmosphere of 5% CO_2_ and 95% humidity. Cells were seeded at a density of 1 × 10⁵ cells/mL and were split every 48 h upon reaching 70% confluence.

### 2.12. Statistical Analysis

After conducting three experiments, descriptive statistics (mean and standard deviation) of the quantitative data were calculated. A mean comparison analysis was performed using the ANOVA test with a 95% confidence level, followed by Tukey’s multiple range test (*p* < 0.05). The statistical analysis was carried out using the SPSS statistical package. Survival data are presented using Kaplan–Meier graphs, and the median lethal dose (LD_50_) was calculated using probit analysis in SPSS version 18.0.

## 3. Results

One advantage of the improved genotypes is that they are composed of up to 95% liquid, a characteristic that facilitates the extraction of metabolite-rich juices. In this regard, the fruits of *S. compositum* demonstrated a higher yield of phenolic acids when extracted from juice compared to extraction from dried fruit biomass (dried at 45 °C) using ethanolic and methanolic solvents (Table 2; Figures S1A–C and S2A–C).

*Sechium* extracts are typically dried in an oven at 45 °C before proceeding with the liquid–liquid extraction process. This drying step helps preserve the plant material and its qualities; however, the type and duration of drying can alter the composition of secondary metabolites (Sm), potentially reducing their concentration. As shown in Table 3, the flavonoid yield was significantly higher when the extraction was performed using fresh fruit biomass compared to dried material.

Table 4 indicates that cucurbitacin B was one of the least degraded compounds following the drying process. However, no other studies have reported similar findings for the other cucurbitacins present *in S. compositum* fruits. Notably, cucurbitacin I showed a 1.4% higher content in the extracts compared to the juice of fresh fruits.

The phytochemical composition of the bitter genotypes of *Sechium*, as well as those resulting from hybridization, is notably complex. This complexity is reflected in the HPLC chromatograms, which show the presence of compounds that have yet to be identified, largely due to the absence of a reference standard. Nevertheless, these unidentified compounds are likely to play a significant role in the plant’s biological activity (Figures S1 and S3A–C). It is important to highlight that certain compounds, such as phloridzin, myricetin, and quercetin, showed substantial degradation in the methanolic extraction, with degradation rates of 93%, 92%, and 96%, respectively, compared to those found in the juice. In contrast, the juice contained less than 40% of the naringenin content and 50% of the phloretin content compared to the methanolic extract.

### 3.1. HD-Victor Hybrid (S. compositum x S. edule *var.* nigrum xalapensis) and *var.* nigrum spinosum

The extract yield was higher in the fruits of HD-Victor (14.8%) compared to the fruits of var. *nigrum spinosum* (6.65%). This difference is primarily due to the lower water content and higher percentage of dry matter in HD-Victor (Table 5). One of the hybrid’s parents, *S. compositum*, has also been noted for high yields, reaching up to 8.6%. Other genotypes, such as Perla Negra™, whose parents are *S. edule* var. *nigrum minor* and *S. edule* var. *amarus sylvestris*, have shown yields of 7.48%. Similarly, the H-387 hybrid (*S. edule* var. *virens levis* and *S. edule* var. *amarus sylvestris*) recorded a yield of 7.59%. The higher yield observed in the hybrids, compared to their parent varieties, can be attributed to the phenomenon of heterosis, also known as hybrid vigor. This occurs when certain traits in the offspring are expressed more strongly than in either parent, resulting in improved performance of the hybrids in terms of yield.

### 3.2. Identification of Secondary Metabolites

High Performance Liquid Chromatography (HPLC) analysis of the HD-Victor extract revealed the presence of flavonoids (Figure S4A,B), cucurbitacins, and phenolic acids (Figure S4C,D). The flavonoids identified included rutin, myricetin, quercetin, naringenin, galangin, and apigenin. In terms of phenolic acids, the analysis detected gallic, chlorogenic, syringic, vanillic, *p*-hydroxybenzoic, caffeic, ferulic, and *p*-coumaric acids, alongside cucurbitacins D, I, B, and E. The flavonoid yields for rutin, myricetin, quercetin, naringenin, galangin, and apigenin were 4.7007, 4.4065, 0.9730, 13.7577, 302.55, and 1.1306 mg g^−1^, respectively, totaling 327.5196 mg g^−1^. For phenolic acids, the quantities were 0.048, 8.9478, 0.3388, 0.5598, 0.4887, 1.3117, 0.1380, and 0.0477 mg g^−1^ for gallic, chlorogenic, syringic, vanillic, *p*-hydroxybenzoic, caffeic, ferulic, and *p*-coumaric acids, respectively. Meanwhile, cucurbitacins D, I, B, and E were quantified at 7.7201, 0.0086, 5.9089, and 0.3283 mg g^−1^, respectively, amounting to a total of 48.1441 mg g^−1^.

### 3.3. Survival of Treated Mice Compared to Healthy Mice

**Figure 2:**
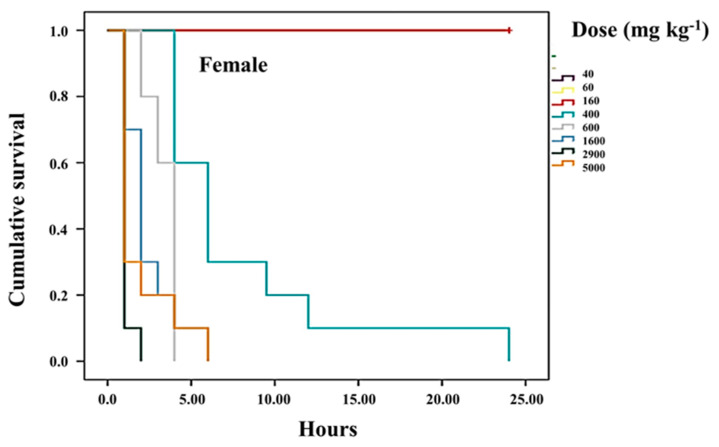
Survival rate of female mice (*n* = 10) treated intraperitoneally with different doses of HD-Victor hybrid extract, evaluated at 24 h post-treatment. Kaplan–Meier graph.

**Figure 3:**
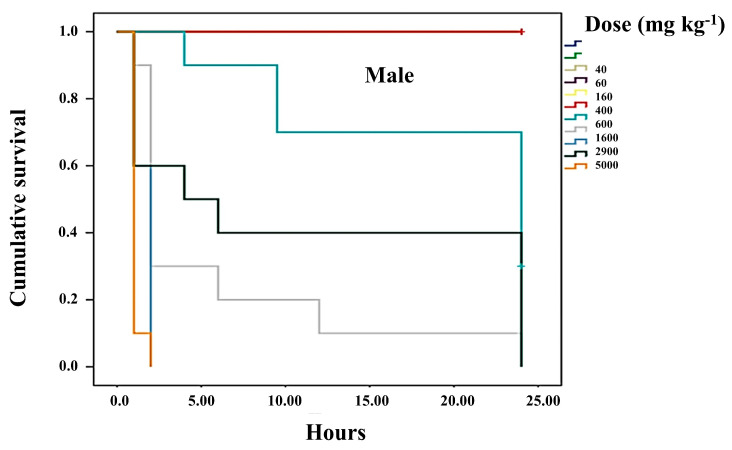
Survival rate of male mice (*n* = 10) treated intraperitoneally with varying doses of the HD-Victor hybrid extract, evaluated 24 h post-treatment. Kaplan–Meier survival plot.

This study confirmed that the median lethal dose (LD_50_) of var. *nigrum spinosum* is greater than 5000 mg kg^−1^ for both male and female CD-1 mice. However, females that were administered high doses of the HD-Victor extract intraperitoneally showed symptoms such as prostration, muscle spasms, and piloerection, with fatalities occurring as early as the first hour and continuing up to 6 h post-administration. At doses of 5000, 2900, and 1600 mg kg^−1^, 100% mortality was recorded. In contrast, doses between 600 and 400 mg kg^−1^ resulted in 50% mortality between the 2nd and 24th hour. Finally, at the lowest dose (160 mg kg^−1^), all females survived up to 24 h after administration (Figure 2). Similarly, males treated with the HD-Victor extract exhibited the same symptoms and experienced 100% mortality at doses of 5000, 2900, and 1600 mg kg^−1^ within the first 24 h post-treatment. The LD_50_ for males was determined to range between 400 and 600 mg kg^−1^, with 50% mortality observed between the 2nd and 24th hour (Figure 3). At doses below 160 mg kg^−1^, all males survived up to 24 h post-treatment.

### 3.4. Determination of the Median Lethal Dose (LD_50_) of the HD-Victor Hybrid

**Figure 4:**
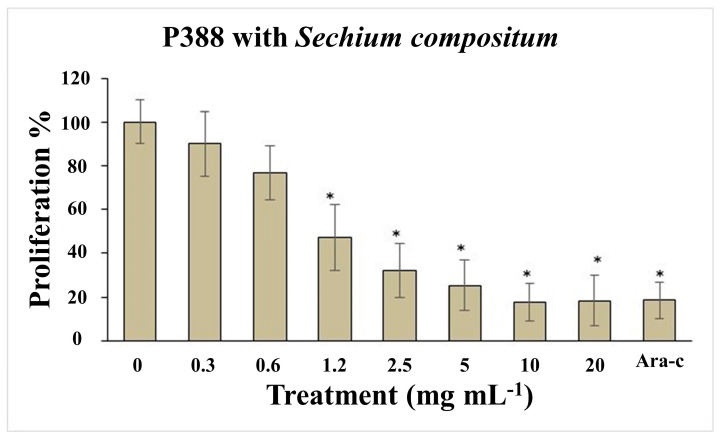
Proliferation effect of the P388 mouse acute myeloid leukemia cell line treated with doses of 0 (phosphate buffer solution), 0.3, 0.6, 1.2, 2.5, 5, 10, and 20 mg mL^−1^ of the methanolic extract of *S. compositum* fruits. Ara-C positive control (5 mg mL^−1^) ± standard deviation. ANOVA followed by Tukey’s test (*p* < 0.01). Asterisks indicate statistically significant differences (*p* < 0.01) between treatments.

**Figure 5:**
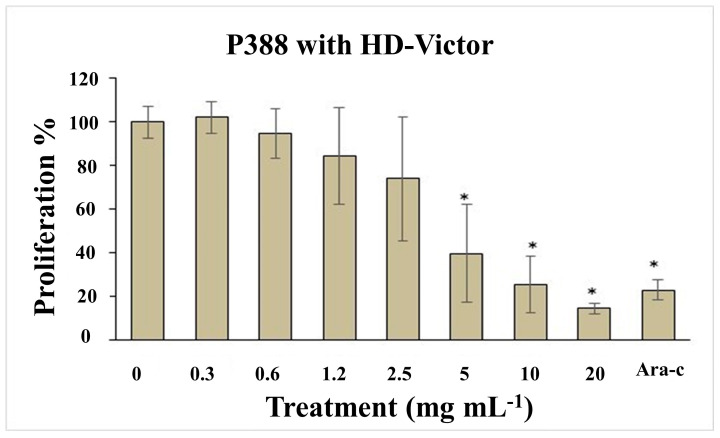
Proliferation effect of the P388 mouse acute myeloid leukemia cell line treated with doses of 0 (phosphate buffer solution), 0.07, 0.15, 0.3, 0.6, 1.2, 2.5, 5, 10, and 20 mg mL^−1^ of the methanolic extract of *S. compositum* fruits. Ara-C positive control (5 mg mL^−1^) ± standard deviation. ANOVA followed by Tukey’s test (*p* < 0.01). Asterisks indicate statistically significant differences (*p* < 0.01) between treatments.

The median lethal dose (LD_50_) was calculated based on the relationship between the intensity of the stimulus and the mortality observed in the mice (probit analysis), following the administration of various doses: 0, 8, 16, 40, 60, 160, 400, 600, 1600, 2900, and 5000 mg kg^−1^. For the HD-Victor extract administered intraperitoneally, the LD_50_ was determined to be 500.14 mg kg^−1^ for females and 517.68 mg kg^−1^ for males. In contrast, for the *S. edule* var. *nigrum spinosum* extract, an LD_50_ of greater than 5000 mg kg^−1^ was recorded for female CD-1 mice. According to recognized toxicity ranges, a dose of 50–500 mg kg^−1^ (oral and cutaneous) is considered moderately toxic, while lower doses (1.0–50 mg kg^−1^) are classified as highly toxic, and higher doses (0.5–5 g kg^−1^) are considered slightly toxic. Regarding the effects on cell proliferation, Figure 4 shows the impact of the methanolic extract of *S. compositum* fruits on the proliferation of the P388 mouse acute myeloid leukemia cell line. A reduction in proliferation was observed at a dose of 0.6 mg mL^−1^, while approximately 80% inhibition of proliferation was recorded at doses of 5.0 mg mL^−1^. This effect was comparable to the positive control, Ara-C (cytarabine), an antineoplastic agent used therapeutically. The mean inhibitory concentration (IC_50_) for proliferation inhibition was calculated to be 2.42 mg mL^−1^. Similarly, Figure 5 illustrates the effect of the methanolic extract of *Sechium* HD-Victor fruits on the proliferation of the P388 cell line. A reduction in proliferation was observed at a dose of 2.5 mg mL^−1^, while an inhibition of approximately 80% was recorded at a dose of 20 mg mL^−1^. As in the previous experiment, this effect was comparable to that of the positive control, Ara-C. The mean inhibitory concentration (IC_50_) for proliferation inhibition was estimated at 5.81 mg mL^−1^.

## 4. Discussion

### 4.1. Sechium Compositum Juice

The juice of fresh *S. compositum* fruits contains significantly higher levels of cucurbitacins, i.e., 99.81% and 96.06% more compared to the ethanolic and methanolic extracts, respectively. This variability in cucurbitacin content has been documented in other species as well. For instance, the methanolic extract of *S. edule* cv. Perla Negra showed cucurbitacin levels of 2540.75 mg kg^−1^, while the H387-07 hybrid exhibited 392.64 mg kg^−1^ [31,32]. Similarly, var. *nigrum spinosum* recorded cucurbitacin levels ranging from 48.14 to 76.82 mg g^−1^. The lower cucurbitacin content in domesticated varieties is likely due to adaptive specialization in response to environmental changes, where the concentration of these compounds has been adjusted as part of the domestication process [33]. In addition to cucurbitacins, flavonoids are another important class of compounds in these plants, and their extraction can be influenced by various factors. The glycosylation of flavonoids, for example, significantly impacts their reactivity and solubility, making them less reactive and more soluble in water [34]. This characteristic plays a crucial role during the extraction process, where methanol often proves to be a more effective solvent than ethanol. Due to its higher polarity, methanol allows for a more efficient extraction of flavonoids and other bioactive compounds [35]. Moreover, the state of the plant material, whether fresh or dried, also affects flavonoid extraction, as drying conditions can alter the chemical composition of the compounds. For example, a previous study [36] investigated the effect of temperature on the total flavonoid content in onion (*Allium cepa* L.), observing that heating the onion powder to 120 °C for 30 min increased flavonoid content, whereas heating it to 150 °C caused a decline. This sensitivity to temperature has also been confirmed in studies on other phenolic compounds. For instance, higher concentrations of total phenols were found in the dried pulp of *Borassus aethiopum* Mart. (African fan palm) fruits at 80 °C compared to lower temperatures [37]. Similarly, research on carrot peel demonstrated that microwave drying at 1200 W resulted in the highest flavonoid content (28.09 mg RE g^−1^ dry sample), while hot air drying produced the lowest flavonoid content (10.81 mg RE g^−1^ dry sample) [38].

On the other hand, cucurbitacins, a distinct class of plant compounds, are oxygenated tetracyclic triterpenoids predominantly produced by Cucurbitaceae species. Known for their bitter taste and toxicity to many organisms, cucurbitacins act as powerful plant defense mechanisms [39]. These triterpenoids are synthesized from mevalonic acid via the isoprenoid pathway, beginning with isopentenyl pyrophosphate (IPP) and continuing through intermediates such as squalene and 2,3-oxidosqualene. The remarkable diversity of triterpenoid structures is primarily determined by the cyclization of 2,3-oxidosqualene by different oxidosqualene cyclases [40]. The efficient extraction of cucurbitacins is closely linked to the solvent used in the process. Methanol’s high polarity plays a key role in maximizing the extraction of these compounds [35]. Cucurbitacins are soluble in a range of organic solvents, including petroleum ether, chloroform, benzene, ethyl acetate, methanol, and ethanol; however, they are insoluble in ether and only slightly soluble in water.

When drying plant material for extraction, optimizing the conditions—such as temperature, drying method, and duration—is crucial to prevent degradation of the compounds. For example, a previous study [41] investigated the effect of drying on cucumbers (*Cucumis myriocarpus* Naudin.) and watermelons (*Cucumis africanus* L.f.), and reported higher cucurbitacin content when the material was dried at 52 °C, with concentrations reaching 9 µg mL^−1^. The study observed a decline in cucurbitacin content as drying temperatures increased. Similarly, other authors [42] compared different drying methods (oven, sun, freezing, and shade) for *Cucumis myriocarpus* and *Cucumis africanus*. The highest concentrations of cucurbitacins A and B were found in oven-dried samples at 52 °C, while other methods resulted in more than an 80% loss of cucurbitacins. The authors concluded that oven drying at the optimal temperature does not harm cucurbitacins A and B.

### 4.2. HD-Victor Hybrid (S. compositum x S. edule *var.* nigrum xalapensis) and S. edule *var.* nigrum spinosum

Currently, almost 25% of all prescription drugs contain one or more active plant-based ingredients [43]. Among these, several antitumor drugs such as vinblastine, irinotecan, and paclitaxel also contain hypoglycemic agents like metformin, which is widely used for diabetes management [44]. In the context of liver injuries, plant-based treatments also play a vital role, with silymarin being a well-known example. Derived from *Silybum marianum* (L.) Gaertn. (milk thistle), silymarin possesses antioxidant properties that help protect against liver damage [45]. Another important hepatoprotective plant is *Sechium edule* (chayote), whose fruits are rich in flavonoids, phenolic acids, and cucurbitacins. These bioactive compounds not only contribute to liver health but also offer a wide range of additional therapeutic benefits, including anti-inflammatory, diuretic [46], antioxidant, antidiabetic, nephroprotective, cardioprotective [47,48], and antitumor activities [49].

Chayote (*Sechium edule*) is not only recognized for its hepatoprotective and therapeutic properties, but also for its rich nutritional profile. Its fruits and seeds contain several essential amino acids, including aspartic acid, glutamic acid, alanine, arginine, cysteine, phenylalanine, glycine, histidine, isoleucine, methionine, proline, serine, tyrosine, threonine, and valine [50]. In addition to these amino acids, eight flavonoids—comprising three C-glycosyl and five O-glycosyl flavones—have been identified in its seeds, along with 20 different gibberellins [32,51]. Furthermore, the leaves of chayote have traditionally been used to treat conditions such as arteriosclerosis and hypertension, and they are also known for their ability to dissolve kidney stones [52].

In addition to its well-documented hepatoprotective and cardioprotective properties, *Sechium edule* has shown promising results in experimental models. For example, authors such as [53] report that different fractions of the ethanol extract from *S. edule* fruits, when administered to Wistar rats with liver damage induced by CCl4, reduced the concentration of liver damage markers (ALT, AST, and FA). Similarly, another study [54] induced myocardial infarction with isoproterenol in Wistar rats and found that the ethanol extracts of *S. edule* fruits exhibited cardioprotective activity. Histological analysis of cardiac tissue from rats treated with the chayote extracts showed less damage compared to untreated controls. However, unlike the present study, previous research often does not specify the variety of *S. edule* used, which limits the reproducibility of these findings. The wide biological variation in *S. edule* influences its Sm content and diversity, leading to differential biological activity [15,49].

Regarding the phytochemical composition of *S. edule* var. *nigrum spinosum*, sources describe the presence of cucurbitacins B, D, and I, alongside phenolic compounds such as gallic, chlorogenic, vanillic, *p*-hydroxybenzoic, caffeic, and *p*-coumaric acids, as well as flavonoids including phlorizidin, naringenin, phloretin, and apigenin [20]. Other genotypes of *S. edule*, such as Perla Negra, also contain cucurbitacins D, I, B, and E, in addition to flavonoids like rutin, myricetin, quercetin, naringenin, phloretin, galangin, and apigenin [32]. Similarly, the hybrid H-387 exhibited cucurbitacins D, I, B, and E, along with rutin, phlorizidin, myricetin, quercetin, naringenin, phloretin, galangin, and apigenin. These findings underscore the fact that cucurbitacins B and E were consistently identified in the three improved variants (HD-Victor, Perla Negra, and H-387), whereas the parent variety, *S. edule* var. *nigrum spinosum*, did not register cucurbitacin E. This suggests that heterosis, or hybrid vigor, could enhance certain nutraceutical properties [55], as cucurbitacins B and E have demonstrated antioxidant capacity and the ability to neutralize free radicals through their glycosides. This highlights the promising potential of cucurbitacin glycosides in the prevention of diseases related to oxidative stress and free radicals, such as liver injury [56].

Authors such as [20] reported concentrations of 0.005, 1.556, 0.018, and 0.290 mg g^−1^ for phlorizidin, naringenin, phloretin, and apigenin, respectively, in the *nigrum spinosum* extract. Meanwhile, the H-387 hybrid extract recorded concentrations of 1.273, 0.0168, 0.889, 0.005, 3.304, 4.616, 21.940, and 0.362 mg g^−1^ for rutin, phlorizidin, myricetin, quercetin, naringenin, phloretin, galangin, and apigenin, respectively. In the case of Perla Negra, another major *S. edule* cultivar, a previous study [32] documented the presence of these flavonoids in lower concentrations: rutin (0.00034 mg g^−1^), myricetin (0.00185 mg g^−1^), quercetin (0.00025 mg g^−1^), naringenin (0.00226 mg g^−1^), phloretin (0.00472 mg g^−1^), galangin (0.00043 mg g^−1^), and apigenin (0.00018 mg g^−1^).

When comparing the hybrids, the concentrations of flavonoids were found to be lower in Perla Negra and *nigrum spinosum*, while the fruits of the H-387 and HD-Victor hybrids demonstrated a greater diversity and higher concentration of flavonoids. Notably, HD-Victor exhibited flavonoid concentrations ten times higher than those of H-387. This finding is particularly significant given the well-documented anti-inflammatory and immunomodulatory properties of flavonoids. Data from in vitro assays suggest that flavonoids can modulate the inflammatory response by interfering with key intracellular signaling pathways, such as NF-κB and MAPK, and by reducing the production of pro-inflammatory interleukins (IL-1, IL-2, IL-6, and IL-8) [57]. In addition to their anti-inflammatory effects, flavonoids also exhibit antioxidant and antibacterial activities. Various flavonoids, including apigenin, galangin, flavones, flavonol glucosides, isoflavones, flavanones, and chalcones, have demonstrated potent antibacterial properties, likely through molecular interactions that disrupt microbial proteins (e.g., adhesins and membrane transport proteins), alter cell membrane permeability, and interfere with cell growth [58]. The antioxidant mechanisms of flavonoids may include suppression of reactive oxygen species (ROS) formation through enzyme inhibition or chelation of trace elements involved in free radical generation, direct ROS scavenging, and the upregulation or protection of antioxidant defenses [59]. Similarly, phenolic acids such as gallic, chlorogenic, vanillic, *p*-hydroxybenzoic, caffeic, and *p*-coumaric acids were reported in the *nigrum spinosum* extract at concentrations of 0.072, 0.823, 0.032, 0.020, 0.091, and 0.032 mg g^−1^, respectively. In comparison, the H-387 hybrid contained 0.056, 4.224, 0.016, 0.087, 0.084, 0.187, 0.064, and 0.029 mg g^−1^ of gallic, chlorogenic, syringic, vanillic, *p*-hydroxybenzoic, caffeic, ferulic, and *p*-coumaric acids, respectively [20]. The Perla Negra cultivar displayed similar concentrations. These results suggest that the HD-Victor hybrid has a higher concentration of phenolic acids, particularly caffeic, ferulic, and chlorogenic acids, which have demonstrated antioxidant and antitumor properties [60,61].

Finally, cucurbitacins were reported in varying concentrations across the different varieties. *Nigrum spinosum* contained cucurbitacins D, I, and B at 0.127, 0.013, and 1.008 mg g^−1^, respectively, while H-387 recorded concentrations of 1.162, 0.088, 1.631, and 0.424 mg g^−1^ for cucurbitacins D, I, B, and E, respectively [20]. In Perla Negra, the concentrations were much lower, with cucurbitacins D, I, B, and E present at 0.00137, 0.00935, 0.0098, and 0.0040 mg g^−1^, respectively [32].

When comparing the hybrids, HD-Victor was found to have a higher concentration of cucurbitacin D and B, while H-387 exhibited a higher concentration of cucurbitacin E, and Perla Negra contained more cucurbitacin I. Although cucurbitacins B, D, and I are known for their extreme toxicity [62], the dose plays a critical role, as the cucurbitacins identified in the fruits of these hybrids and in var. *nigrum spinosum* have demonstrated cytotoxic and antitumor activity [20,49,63]. The higher phytochemical content in HD-Victor suggests it may have a strong hepatoprotective effect, especially due to its high rutin and naringenin content. Both compounds are known to inhibit oxidative stress, regulate transforming growth factor (TGF-β), and prevent the transdifferentiation of hepatic stellate cells (HSCs), which is crucial in liver injury prevention [64,65]. While *Sechium edule* has already been reported to have hepatoprotective effects [53], the superior rutin and naringenin levels in the HD-Victor hybrid make it a potentially better candidate for treating liver injuries.

Considering the biological variation within its infraspecific complex, *S. edule* is thought to exhibit differential biological activity depending on the variety. For example, a previous study [49] observed differential cytotoxic effects of eight varieties on various cancer cell lines, noting that the *albus dulcis*, *albus levis*, *amarus sylvestris*, *nigrum spinosum*, and *nigrum xalapensis* varieties inhibited the proliferation of the P388 murine monocytic leukemia cell line and L929 murine pulmonary fibrosarcoma cells. Specifically, *nigrum spinosum* displayed antiproliferative effects against HeLa and L-929 human cervical cancer cells and reduced the viability of WEHI-3, a murine myelomonocytic leukemia cell line [66]. Additionally, another study [67] reported differences in the antioxidant profiles of three *S. edule* varieties, with variety 845 showing the best in vitro antioxidant profile. However, in the same study, higher doses of strain 853 were found to regulate lipid peroxidation in Sprague Dawley rats.

Finally, the differences identified in terms of the lower toxicity level observed in the HD-Victor hybrid extract compared to *S. compositum*, one of its parental varieties, could be explained not only by its differential phytochemical composition, but also by additional factors that deserve consideration. For example, the hybrid’s complex chemical matrix could favor interactions between compounds that modulate its toxicity. Certain flavonoids and phenolic acids present in higher proportions in HD-Victor, such as naringenin and chlorogenic acid, have been shown to act as antagonists of cytotoxic molecules, reducing their toxic effects in biological systems [35,40]. Although cucurbitacins, a key component in *S. compositum*, possess significant bioactive properties, their excessive concentration can be highly toxic. In this case, the HD-Victor hybrid presents reduced levels of specific cucurbitacins, such as cucurbitacin I, which could contribute to its lower toxicity. Additionally, hybridization introduces genetic reorganization that can impact the expression of genes related to the biosynthesis of secondary metabolites. This phenomenon, known as heterosis, could explain the reduction in the production of specific cytotoxic compounds or the synthesis of protective metabolites in the hybrid [42,68]. Finally, the extract matrix of the HD-Victor hybrid, compared to *S. compositum*, might include additional secondary metabolites that mitigate toxic effects, such as antioxidants or anti-inflammatory compounds. Previous studies have demonstrated that the combination of secondary metabolites in a specific matrix can significantly influence the biological and toxicological properties of extracts [39]. Although phytochemical data provide an initial basis for explaining these differences, it would be interesting for future studies to analyze the transcriptomic and metabolomic profiles of the hybrid and its parentals. This would allow the identification of key genes involved in metabolite synthesis and potential interactions among them that contribute to its lower toxicity.

## 5. Conclusions

The results of this study highlight the potential of *Sechium* genotypes as sources of bioactive secondary metabolites with significant therapeutic applications. HPLC analysis revealed that the HD-Victor hybrid exhibited the highest concentrations of phenolic acids, flavonoids, and cucurbitacins, surpassing both parental varieties, and demonstrated significantly lower toxicity compared to *S. compositum*. This reduced toxicity is likely a result of its distinct phytochemical profile, which includes higher levels of protective flavonoids and phenolic acids, as well as potential synergistic interactions between metabolites and heterosis effects. Moreover, the degradation of over 90% of phenolic acids and cucurbitacins during the drying process of *S. compositum* emphasizes the need to refine extraction methods to preserve these valuable compounds. The HD-Victor hybrid stands out as a particularly promising genotype for future in vitro and in vivo evaluations due to its superior metabolite content and favorable safety profile. These findings provide a robust foundation for advancing the development of *Sechium* spp. as nutraceutical and therapeutic resources, while also underscoring the importance of optimized processing methods to enhance their bioactive potential.

## Figures and Tables

**Table 1:** Bitter and edible genotypes of *Sechium* spp., as food source and metabolites.

Name	Genotypes	Varietal Distinction	Flavor
631-12	*Sechium edule var. nigrum minor*	variety	bitter
632-12	*S. compositum* (Donn. Sm.) C. Jeffrey	wild type	bitter
633-12	*S. compositum x S. edule* var*. nigrum maxima*	hybrid	bitter
635-12	*S. compositum x S. edule* var*. nigrum spinosum*	hybrid	bitter
636-12	*S. chinantlense* Lira & Chiang	wild type	bitter
639-12	*S. edule* var*. amarus sylvestris x S. edule* var*. virens levis*	639-12-H-D	bitter
641-12	*S. edule* var*. nigrum xalapensis x S. edule* ve*r. amarus sylvestris*	hybrid	bitter
643-12	*S. compositum x S. edule var. nigrum xalapensis*	H-D-Victor	bitter
H387	*S. edule* var*. amarus sylvestris x S. edule* var*. virens levis*	H-387	bitter
Campiña	*Sechum edule* (Jacq) Sw.	Campiña Reg. 0855	edible
Cañitas	*Sechum edule* (Jacq) Sw.	Cañitas Reg. 0856	edible
Ventlali	*Sechum edule* (Jacq) Sw.	Ventlali Reg. 0857	edible

The identifiers included in the “Name” column correspond to unique codes assigned to *Sechium* spp. genotypes in the germplasm bank. These codes are used for the classification and tracking of the genotypes evaluated in the present study.

**Table 2:** Phenolic acid content in methanolic and ethanolic extracts and fruit juice of *Sechium compositum*.

Phenolic Acids (mg kg^−1^)			
Compound	Methanolic Extract (Dry Biomass)	Ethanolic Extract (Dry Biomass)	Juice (Fresh Biomass)
gallic acid	42.82 ± 0.022 c	62.35 ± 0.021 b	115.44 ± 0.034 a
chlorogenic acid	3.56 ± 0.01 b	0. 00 ± 0.0 c	451.20 ± 0.024 a
syringic acid	1.94 ± 0.01 b	1.93 ± 0.012 b	12.86 ± 0.026 a
vanillinic acid	0.00 ± 0.0 b	1.01 ± 0.013 b	19.61 ± 0.032 a
*p*-hydroxybenzoic acid	2.42 ± 0.031 b	0.82 ± 0.013 b	36.66 ± 0.025 a
caffeic acid	1.31 ± 0.011 b	2.88 ± 0.03 b	76.89 ± 0.015 a
ferulic acid	3.32 ± 0.023 a	1.84 ± 0.013 ab	0.00 ± 0.0 b
*p*-coumaric acid	0.68 ± 0.01 b	0.50 ± 0.01 b	19.51 ± 0.0.31 a
Sum of quantified compounds	56.05	71.34	732.16

Values with the same letters within rows indicate no significant differences (Tukey, *p* < 0.05, ±standard error). The term “sum” refers to the quantified compounds listed in the table and does not represent the total phenolic or flavonoid content of the extract.

**Table 3:** Content of flavonoids in methanolic and ethanolic extracts and the fruit juice of *Sechium compositum*.

Flavonoids (mg kg^−1^)			
Compound	Methanolic Extract (Dry Biomass)	Ethanolic Extract (Dry Biomass)	Juice (Fresh Biomass)
rutin	42.45 ± 0.001 b	1.49 ± 0.002 c	62.70 ± 0.0023 a
phloridzin	6.20 ± 0.003 b	5.18 ± 0.001 b	85.46 ± 0.0011 a
mirectin	11.01 ± 0.004 b	0.59 ± 0.003 b	145.83 ± 0.00151 a
quercetin	0.56 ± 0.001 b	1.90 ± 0.004 b	15.37 ± 0.0014 a
naringenin	303.69 ± 0.005 a	58.51 ± 0.0012 c	182.70 ± 0.002 b
phloretin	51.84 ± 0.0021 a	10.39 ± 0.0032 a	25.44 ± 0.0031 a
galangin	6.55 ± 00.27 a	5.23 ± 0.0026 a	4.08 ± 0.0015 a
Sum of quantified compounds	422.30	83.29	521.59

Values with the same letters within rows indicate no significant differences (Tukey, *p* < 0.05, ±standard error). The term “sum” refers to the quantified compounds listed in the table and does not represent the total phenolic or flavonoid content of the extract.

**Table 4:** Cucurbitacin content (mg kg^−1^ of fresh matter) in methanolic and ethanolic extracts and chayote fruit juice of *Sechium compositum*.

Cucurbitacins (mg kg^−1^)			
Compound	Methanolic Extract (Dry Biomass)	Ethanolic Extract (Dry Biomass)	Juice (Fresh Biomass)
cucurbitacin D	17.24 ± 0.001 a	0.11 ± 0.001 b	7.93 ± 0.0031 ab
cucurbitacin I	25.45 ± 0.0031 b	0.121 ± 0.003 b	1723.39 ± 0.0021 a
cucurbitacin B	1.03 ± 0.0042 a	0.50 ± 0.003 a	1.888 ± 0.0016 a
cucurbitacin E	24.99 ± 0.0012 a	2.57 ± 0.0014 b	15.24 ± 0.0014 a
Sum of quantified compounds	68.78	3.31	1748.45

Values with the same letters within rows indicate no significant differences (Tukey, *p* < 0.05, ±standard error). The term “sum” refers to the quantified compounds listed in the table and does not represent the total phenolic or flavonoid content of the extract.

**Table 5:** Yield in dry weight and extract of *S. edule* var*. nigrum spinosum* and HD-Victor hybrid, from horticulturally mature fruits.

Variable	*S. edule* var*. nigrum spinosum*	HD-Víctor
Fresh weight (kg)	80.00	15.00
Dry weight (kg)	5.24	1.56
Water (%)	93.44	89.60
Dry matter yield (%)	6.55	10.40
Extract yield (%)	6.65	14.80

## Data Availability

The original contributions presented in the study are included in the article/Supplementary Material, further inquiries can be directed to the corresponding author.

## Supplementary Materials

The following supporting information can be downloaded at: https://www.mdpi.com/article/10.3390/life15010015/s1; Figure S1: Chromatograms of the ethanolic extract of *Sechium compositum* fruits obtained by HPLC. A: Phenolic acids, B: Flavonoids and C: Cucurbitacins; Figure S2: Chromatograms of the methanolic extract of *Sechium compositum* fruits obtained by HPLC. A: Phenolic acids, B: Flavonoids and C: Cucurbitacins; Figure S3: Chromatograms of *Sechium compositum* fruit juice obtained by HPLC. A: Phenolic acids, B: Flavonoids and C: Cucurbitacins; Figure S4: A,B. Flavonoid chromatograms. C,D: chromatograms of cucurbitacins and phenolic acids present in the extract of the hybrid HD-Victor; Table S1. Calibration curves for phenolic acids and flavonoids generated using external standards. Linear regression analysis was performed, and the resulting equations demonstrated good linearity (R^2^ > 0.995); Table S2. The LOD and LOQ values for each compound analyzed; Table S3. The retention times of each compound were determined under optimized HPLC conditions and are presented below; Table S4. Recovery percentage of identified compounds.
