# Supplementary material for: Genotypes of Sechium spp. as a Source of Natural Products with Biological Activity
Source: Life (Basel). 2024 Dec 27;15(1):15. doi: 10.3390/life15010015 (PMC11766598; doi:10.3390/life15010015)
Supplement: Supplementary file 1 [file life-15-00015-s001.zip › life-3294442-supplementary.pdf]

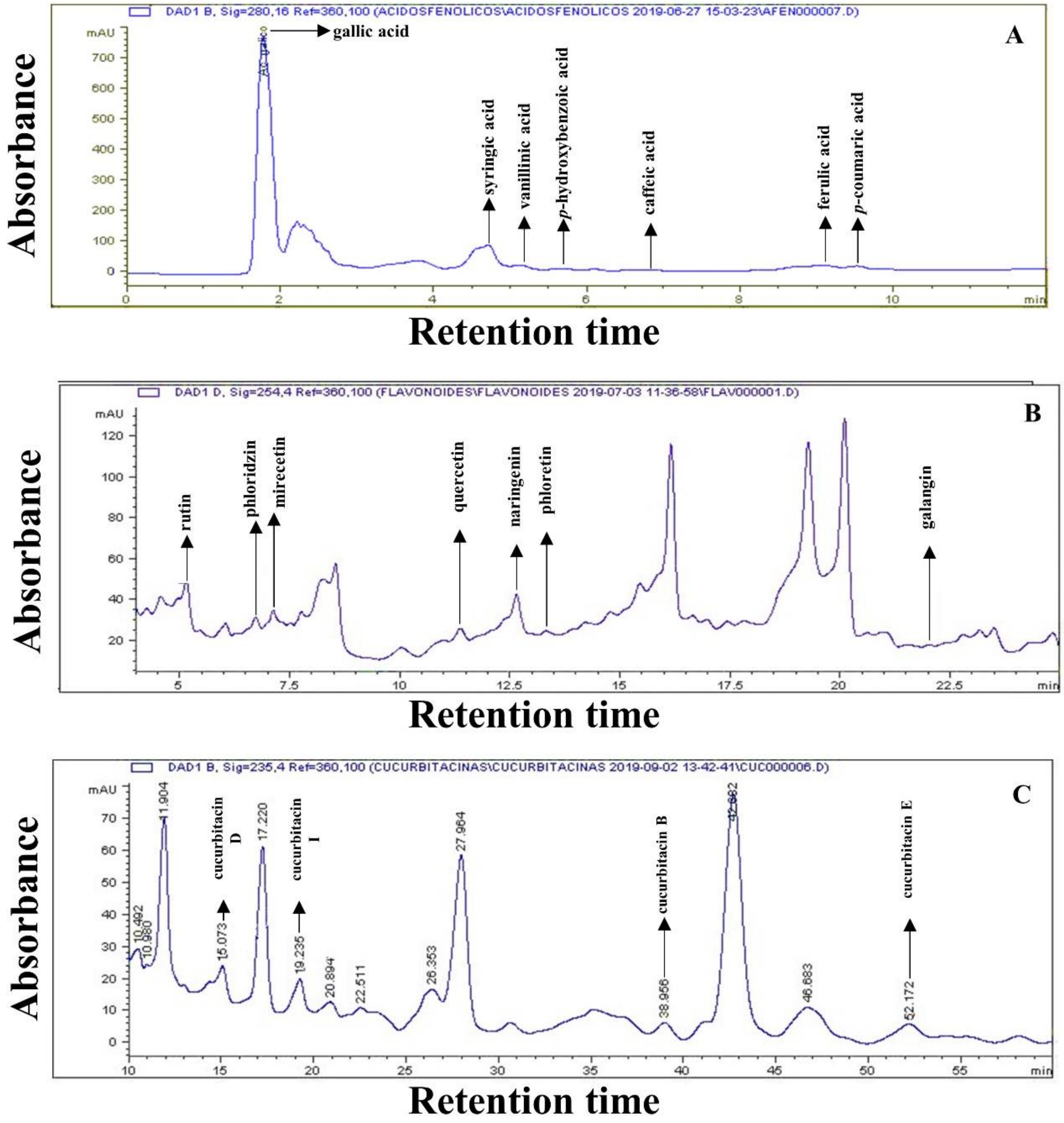

Supplementary Figure S1. Chromatograms of the ethanolic extract of *Sechium compositum* fruits obtained by HPLC. A: Phenolic acids, B: Flavonoids and C: Cucurbitacins.

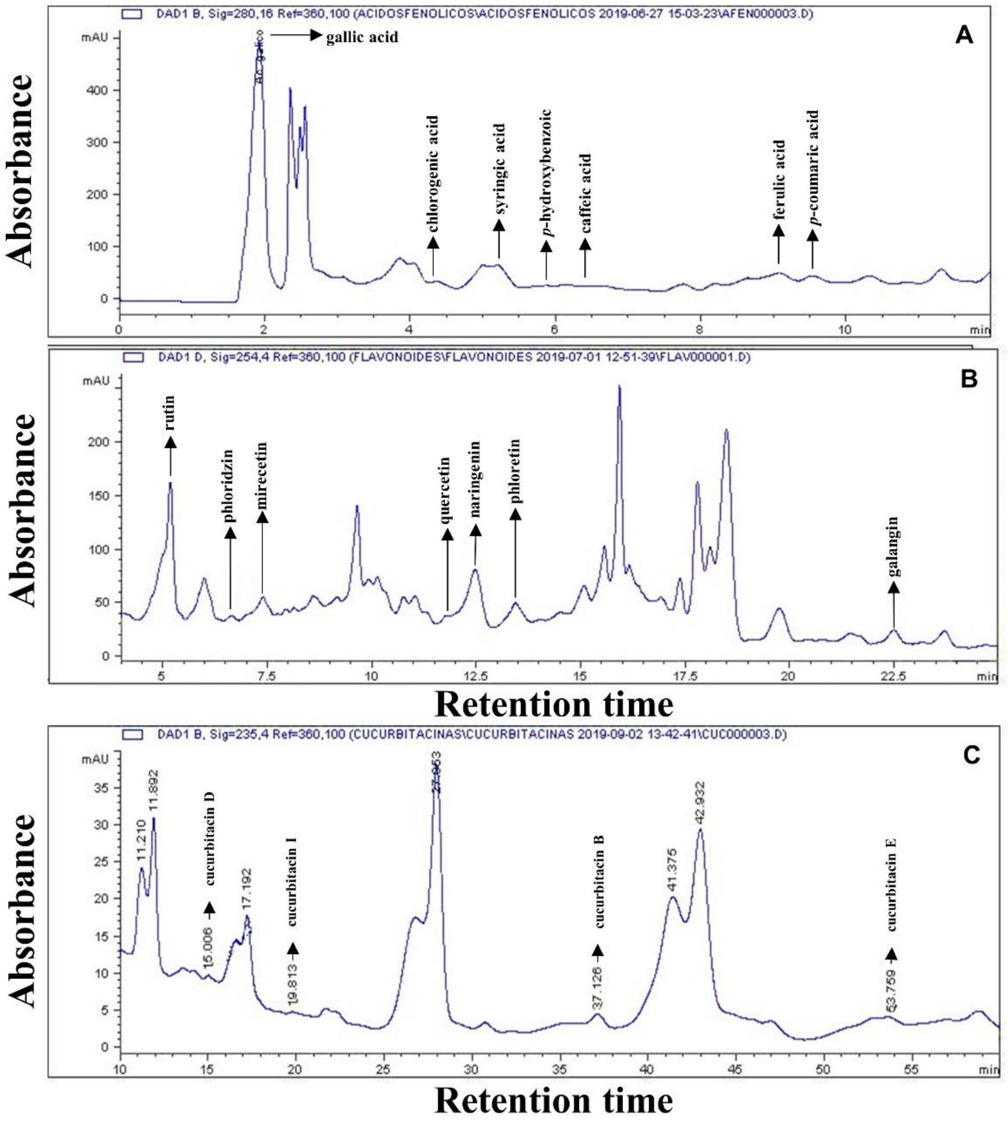

Supplementary Figure S2. Chromatograms of the methanolic extract of *Sechium compositum* fruits obtained by HPLC. A: Phenolicacids, B: Flavonoids and C: Cucurbitacins.

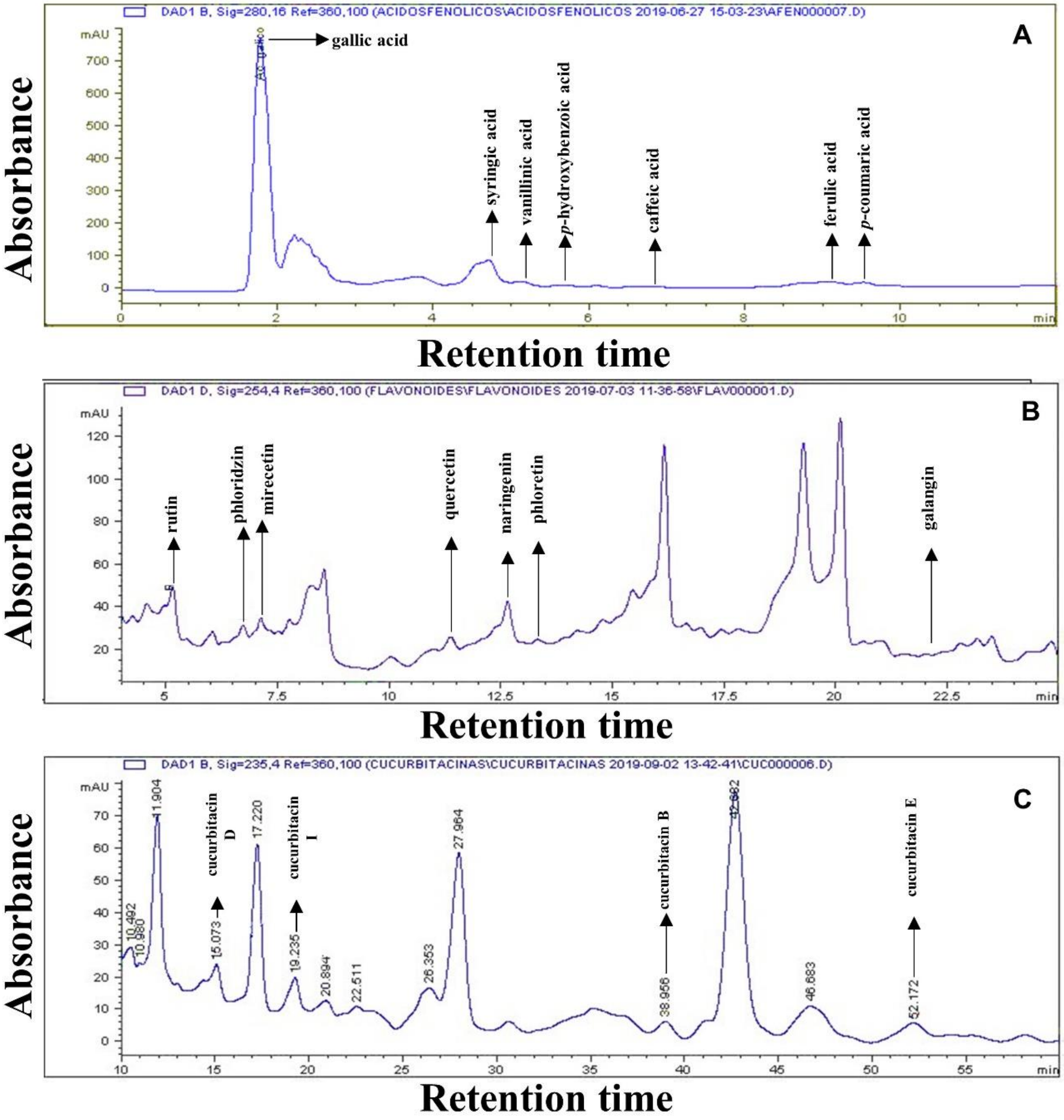

Supplementary Figure S3. Chromatograms of *Sechium compositum* fruit juice obtained by HPLC. A: Phenolic acids, B: Flavonoidsand C: Cucurbitacins.

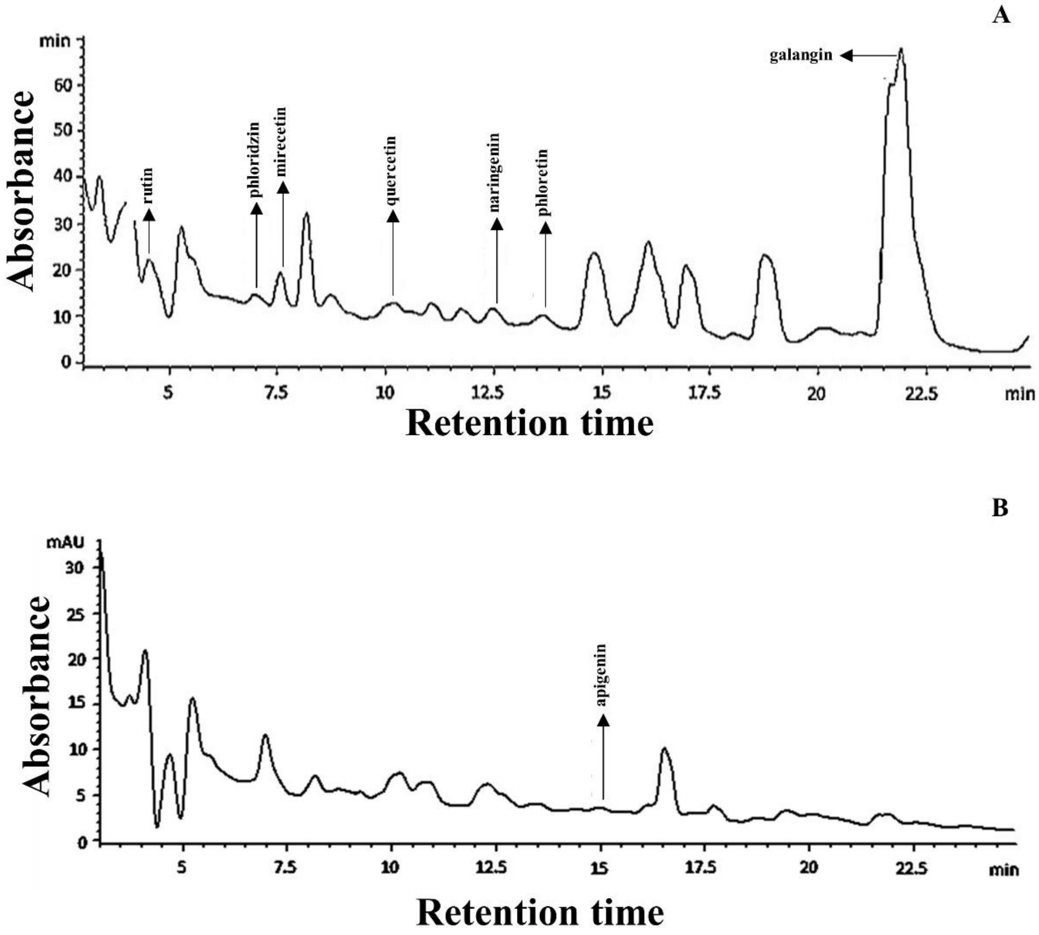
A
galangin
rutin
phloridzin
mirecetin
quercetin
naringenin
phloretin
Absorbance
min
0
10
20
30
40
50
60
5
7.5
10
12.5
15
17.5
20
22.5
min
Retention time
B
mAU
0
5
10
15
20
25
30
apigenin
Absorbance
5
7.5
10
12.5
15
17.5
20
22.5
min
Retention time

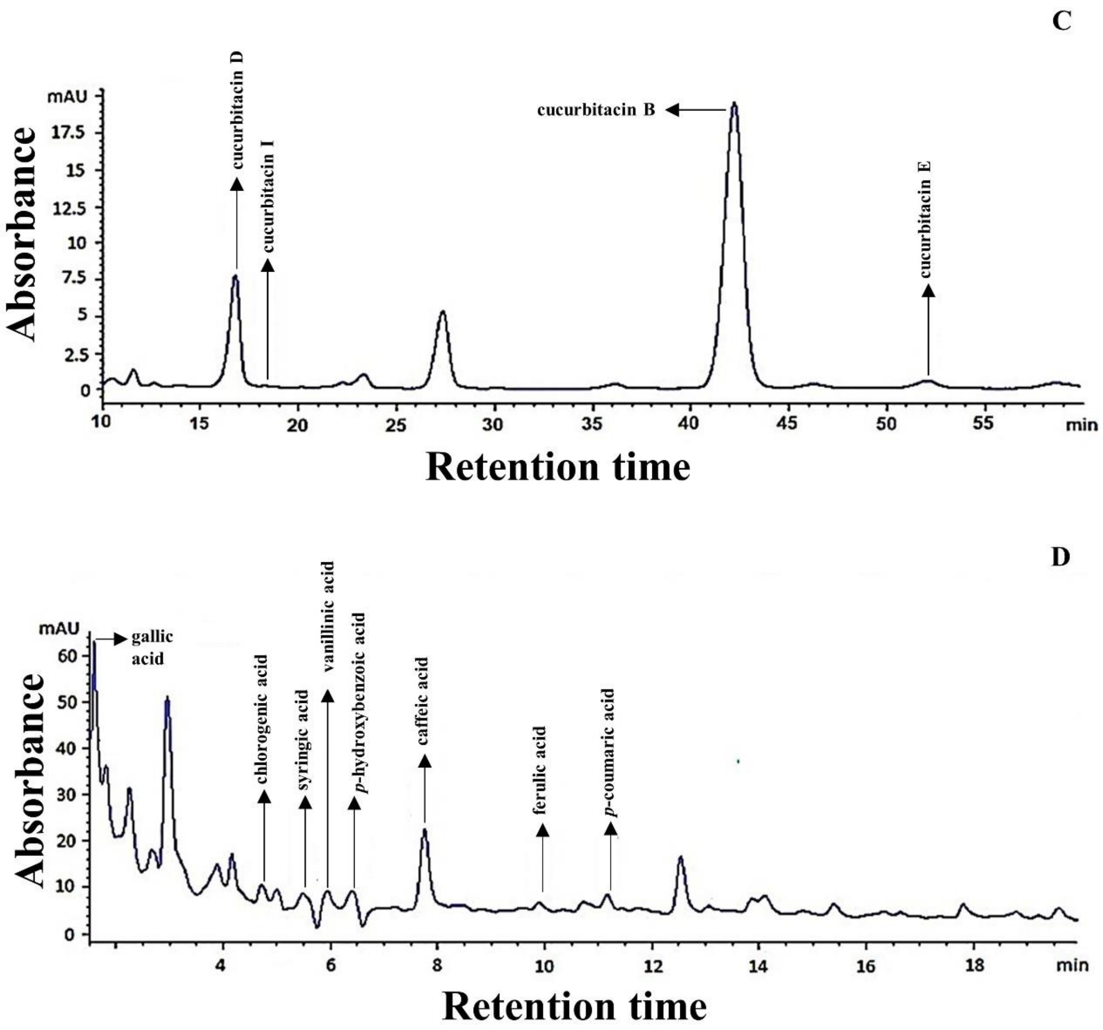

Supplementary figure S4. A-B. Flavonoid chromatograms. C-D: chromatograms of cucurbitacins and phenolic acids present in the extractof the hybrid HD-Victor.

Supplementary table S1. Calibration curves for phenolic acids and flavonoids generated using external standards. Linear regression analysis was performed, and the resulting equations demonstrated good linearity ($R^2 > 0.995$).

Compound	Calibration Equation	R^2
**Phenolic Acids**		
Gallic acid	$y = 12045x + 205.12$	0.9979
Chlorogenic acid	$y = 9823x + 185.45$	0.9975
Syringic acid	$y = 10210x + 152.24$	0.9980
Vanillic acid	$y = 11045x + 198.42$	0.9973
p-Hydroxybenzoic acid	$y = 11480x + 140.65$	0.9970
Caffeic acid	$y = 10720x + 210.15$	0.9968
Ferulic acid	$y = 9500x + 187.53$	0.9976
p-Coumaric acid	$y = 9705x + 172.88$	0.9971
**Flavonoids**		
Rutine	$y = 11200x + 201.23$	0.9978
Phloridzin	$y = 10800x + 230.32$	0.9974
Myricetin	$y = 10780x + 215.18$	0.9976
Quercetin	$y = 11810x + 182.64$	0.9972
Naringenin	$y = 9400x + 250.89$	0.9980
Phloretin	$y = 11000x + 212.67$	0.9975
Galangin	$y = 10150x + 202.56$	0.9973
**Cucurbitacins**		
Cucurbitacin B	$y = 8950x + 180.64$	0.9969
Cucurbitacin D	$y = 9200x + 190.42$	0.9975
Cucurbitacin E	$y = 9100x + 185.65$	0.9973
Cucurbitacin I	$y = 8800x + 175.45$	0.9978

Supplementary table S2. The LOD and LOQ values for each compound analyzed.

Compound	LOD (mg/mL)	LOQ (mg/mL)
Gallic acid	0.05	0.01
Chlorogenic acid	0.07	0.02
Syringic acid	0.06	0.01
Vanillic acid	0.05	0.01
p-Hydroxybenzoic acid	0.06	0.01
Caffeic acid	0.08	0.02
Ferulic acid	0.07	0.02
p-Coumaric acid	0.06	0.01
Rutine	0.05	0.01
Phloridzin	0.07	0.02
Myricetin	0.06	0.01
Quercetin	0.06	0.01
Naringenin	0.08	0.02
Phloretin	0.07	0.02
Galangin	0.06	0.01
Cucurbitacin B	0.05	0.02
Cucurbitacin D	0.07	0.02
Cucurbitacin E	0.06	0.01
Cucurbitacin I	0.06	0.01

Supplementary table S3. The retention times of each compound were determined under optimized HPLC conditions and are presented below.

Compound	Retention Time (min)
Gallic acid	1.903
Chlorogenic acid	4.209
Syringic acid	4.408
Vanillic acid	5.570
p-Hydroxybenzoic acid	5.673
Caffeic acid	6.647
Ferulic acid	9.207
p-Coumaric acid	9.506
Rutine	5.185
Phloridzin	6.667
Myricetin	7.395
Quercetin	11.407
Naringenin	12.507
Phloretin	13.397
Galangin	22.345
Cucurbitacin B	38.956
Cucurbitacin D	15.073
Cucurbitacin E	52.172
Cucurbitacin I	19.235

Supplementary table S4. Recovery percentage of identified compounds.

Compound	Recovery (%)
Gallic acid	89.5 ± 2.1
Chlorogenic acid	88.0 ± 1.8
Syringic acid	90.2 ± 1.6
Vanillic acid	87.8 ± 2.4
p-Hydroxybenzoic acid	85.6 ± 1.9
Caffeic acid	88.4 ± 2.2
Ferulic acid	86.5 ± 1.7
p-Coumaric acid	87.1 ± 2.5
Rutine	91.2 ± 1.3
Phloridzin	89.8 ± 2.0
Myricetin	86.4 ± 1.8
Quercetin	88.9 ± 2.1
Naringenin	83.5 ± 1.5
Phloretin	87.8 ± 1.9
Galangin	87.3 ± 2.2
Cucurbitacin B	90.1 ± 1.0
Cucurbitacin D	90.7 ± 1.7
Cucurbitacin E	92.2 ± 2.2
Cucurbitacin I	91.0 ± 1.8
